# Possible Correlation between Hypomelanosis of Ito and Wilms' Tumor

**DOI:** 10.1155/2018/5938120

**Published:** 2018-07-16

**Authors:** Daniella Bello-Germino, Rasmey Chhin, Thu Tran, Tetyana L. Vasylyeva

**Affiliations:** Department of Pediatrics, Texas Tech University Health Sciences Center, Amarillo, TX, USA

## Abstract

Hypomelanosis of Ito is a neurocutaneous disorder characterized by skin manifestations in a characteristic pattern associated with musculoskeletal and central nervous system symptoms. Our patient was diagnosed with Wilms' tumor stage I at age two and was also found to have distinct streaked areas of skin hyper- and hypopigmentation suggestive of Hypomelanosis of Ito. We believe that our patient's clinical diagnoses of Hypomelanosis of Ito and Wilms' tumor are interlinked. The connecting factor is yet to be identified. Our patient does not have a deletion of 11p13 associated with a defect in WT1, the Wilms' tumor suppressor gene. As such, it is quite possible that what made her more susceptible to the development of Wilms' tumor was her Hypomelanosis of Ito, which is implicated in a number of other organ dysfunctions.

## 1. Introduction

Hypomelanosis of Ito, described in 1952 as incontinentia pigmenti achromians, is a rare neuroectodermal disorder characterized by mainly hypopigmented and in some cases hyperpigmented anomalies following the Blaschko lines associated with neurological, ocular, and musculoskeletal abnormalities. The prevalence of this disorder has been difficult to estimate because of the paucity of reported cases in the literature, but it is reported to be around 1/1,000 to 3/10,000 children affecting both sexes equally. Manifestations include hypopigmented areas following the lines of normal cell development in the skin that might present as bilateral or unilateral lesions corresponding to the normal distribution and migration of the two distinct cell clones with different pigment potential. These lesions tend to appear within the first year of life in three-fourths of patients and seem to be more prominent in the flexor surfaces of the limbs and the ventral surface of the trunk [1].

Extradermal manifestations are also present. Neurological disorders, primarily seizures, and mental retardation have been found in 70% of patients. More predominantly, 94% of patients have some musculoskeletal abnormalities including limb discrepancies, scoliosis, and abnormalities of the digits such as syndactyly, polydactyly, and bifid thumb. Abnormalities involving other systems have been reported including head and face anomalies, cardiac anomalies like ventricular or atrial septal defects, and genital and reproductive organ anomalies including precocious puberty, cryptorchidism, micropenis, hypospadias, urethral duplications, and nephritis, among others.

Hypomelanosis of Ito is caused by chromosomal mosaicism that can occur in a variety of autosomal diseases or may be associated with an alteration of a sex chromosome. However, the genetic mechanism and pathogenesis is not yet completely understood. In the majority of cases, there are generally no affected relatives and the syndrome seems to occur sporadically [2]. Epidemiological data of this disease are very limited. It is the third most common cause of neurocutaneous syndromes.

The prognosis is not related to the degree of mosaicism. Instead, the more systemic complications are reported, the worse the prognosis. When cutaneous involvement is the only manifestation, the prognosis is excellent.

## 2. Case

Our patient was born term without complications and had an unremarkable stay in newborn nursery. At age 2, our patient was noted by her parents to have an abdominal “knot” on her right side. In addition, she had unremitting fevers. She was seen at an outside emergency department, and a CBC was found to have WBC 23,300, H/H 10.1/28.9, platelets 416,000, neutrophils 68%, bands 14%, and lymphocytes 12%. AST and ALT were elevated at 117 and 231, respectively. She was transferred to our hospital for concerns of an oncological diagnosis.

At our facility, physical exam of the abdomen revealed a fixed, nontender mass on the patient's right side with slight abdominal distension. An initial CT with contrast identified a heterogeneous mass measuring 8.7 × 7.2 × 8.0 cm. She underwent a right nephrectomy, and pathology reports identified a tumor measuring 9.2 × 7.0 × 6.5 cm with blastemal, tubular, and mesenchymal components confined to the kidney. In close proximity was found to be another tumor 1.0 cm in diameter also without lymphatic or vascular invasion beyond the kidney.

Our patient was diagnosed with nephroblastoma, or Wilms' tumor, of the right kidney. Staging of the mass and lymph nodes confirmed status as stage 1. Given a favorable histology, she did not require chemotherapy or radiation therapy. Since her nephrectomy, she has been in remission. Annual ultrasounds have confirmed that there is no tumor recurrence. Nearly seven years into her initial diagnosis, a urinalysis revealed hematuria, 3–10 RBCs, concerning for possible relapse or underlying renal abnormality. She was referred to the nephrology service for further evaluation.

Upon presentation to our nephrology service, a renal ultrasound, with surgical absence of the right kidney, was unremarkable. Urine culture and urine calcium to creatinine ratio was within normal limits. Repeat urinalysis did not reveal hematuria. A urine dipstick, however, exhibited a small number of leukocytes and moderate hematuria suggestive of an underlying urinary tract infection, and as such, our patient was treated with an antibiotic course.

**Figure 1:**
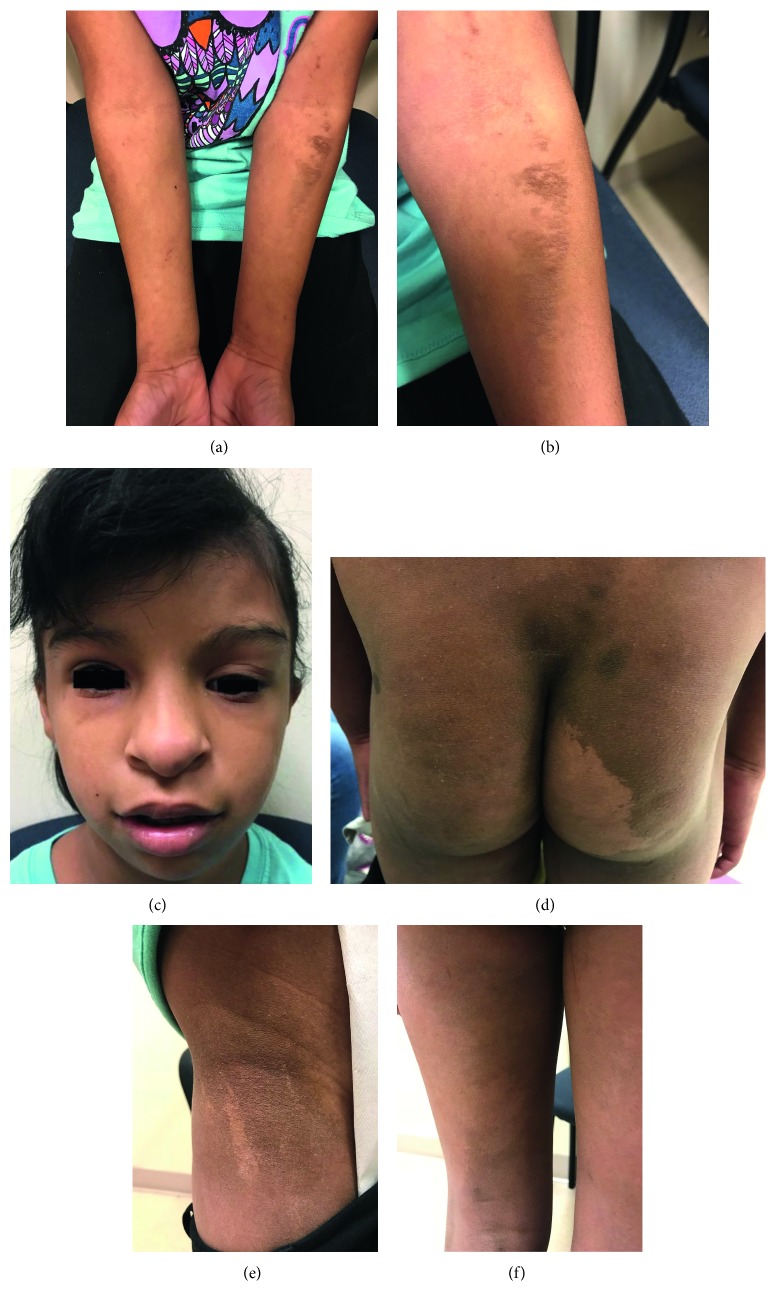
(a) Bilateral forearms. (b) Close view of left forearm. (c) Face. (d) Buttocks. (e) Left waist. (f) Posterior left thigh.

Besides the abdominal exam findings, she had very impressive hyperpigmented lesions scattered across her body as demonstrated in Figure 1. On her left cheek is a prominent area of streaked hypopigmentation. The streaks are also seen on her arms, legs, and back. In addition, she exhibits “crossed” hemihypertrophy with microcephaly on the left side of her face, including microphthalmia of her left eye and a notably larger left leg when compared to the right leg. Our patient also had developmental delay requiring speech and occupational therapy. She also presented with weakness of her left eye musculature, which could very well be related to her left eye being smaller than her right eye, requiring concomitant vision therapy.

Genetic testing had been performed due to concerns for WAGR syndrome, which is characterized by Wilms' tumor, aniridia, genitourinary abnormalities, and mental retardation or developmental delay, two of which our patient had [3]. Wilms' tumor is oftentimes associated with a deletion of 11p13, which houses WT1, the Wilms' tumor suppressor gene [4]. Our patient's chromosomal karyotyping and FISH testing did not reveal any conclusive diagnosis.

## 3. Discussion

Hypomelanosis of Ito is fairly new in terms of being recognized, studied, and understood. Most commonly, there are related neurological and musculoskeletal anomalies. This may very well be because of the easily identified physical dysfunctions, such as developmental delays associated with these anomalies. Renal dysfunctions have not often been described in the literature. The mosaicism that most often causes Hypomelanosis of Ito likely results in the disruption of other cell lines that ultimately creates disturbances in a patient's physiology.

Given our patient's dermatologic findings, she was clinically diagnosed with Hypomelanosis of Ito, though it was never confirmed through genetic testing. Her Wilms' tumor could represent a propensity towards renal dysfunction given her underlying dermatologic diagnosis. Of note, our patient has not presented with any neurological manifestations other than developmental delay. She is receiving therapies to improve her quality of life. However, we must be cognizant that she may develop different, more debilitating conditions as potential complications of her disease, and should be regularly followed by a healthcare provider. A CT or MRI of her brain could identify possible dysfunctions but is not warranted in a neurologically stable patient.

Our patient's left leg being notably bigger than her right leg represents a limb discrepancy previously identified in other patients with Hypomelanosis of Ito [5]. In her case, this is more a cosmetic issue and does not interfere with her activities of daily living.

We believe her Hypomelanosis of Ito reflects a genetic error that also increased her propensity to develop Wilms' tumor. Her karyotype and FISH testing did not confirm any chromosomal abnormalities. However, mosaicism is the most common culprit in Hypomelanosis of Ito and would be identified through karyotyping. If a patient presents with striking systemic features, for instance, neurologic dysfunctions such as seizures and mental retardation in addition to streaked skin findings, karyotyping should be performed [6]. Our patient's diagnosis was made clinically, but there is without a doubt an underlying genetic disturbance we just have not yet uncovered.

## 4. Conclusion

We present a patient with Hypomelanosis of Ito who also had Wilms' tumor. We suspect there is a connection between the two diagnoses with Hypomelanosis of Ito causing some kind of super influence on the genome leading to an increased susceptibility to renal pathology. Given our patient's findings, we suggest that should other patients present with Hypomelanosis of Ito, it would be beneficial to consider renal dysfunctions as a potential correlated diagnosis. If recognized early, the risk of morbidity and mortality may be reduced. It may also be of benefit for other patients with Hypomelanosis of Ito to be followed by a nephrology service earlier given the potential for a renal oncological disease.
